# Circular RNA, microRNA and Protein Profiles of the Longissimus Dorsi of Germany ZIKA and Sichuan White Rabbits

**DOI:** 10.3389/fgene.2021.777232

**Published:** 2021-12-24

**Authors:** Xiangyu Zhang, Cuixia Zhang, Chao Yang, Liangde Kuang, Jie Zheng, Li Tang, Min Lei, Congyan Li, Yongjun Ren, Zhiqiang Guo, Yang Ji, Xiaodong Deng, Dengping Huang, Gaofu Wang, Xiaohong Xie

**Affiliations:** ^1^ Sichuan Animal Sciences Academy, Chengdu, China; ^2^ Animal Breeding and Genetics Key Laboratory of Sichuan Province, Chengdu, China; ^3^ Chongqing Academy of Animal Sciences, Chongqing, China

**Keywords:** circular RNA, miRNA, muscle, longissimus dorsi, rabbit

## Abstract

Due to the dietetic properties and remarkable nutritive value of rabbit meat, its industry is increasing rapidly. However, the association between circular RNAs, microRNAs, and proteins and muscle fiber type, and meat quality of rabbit is still unknown. Here, using deep sequencing and iTRAQ proteomics technologies we first identified 3159 circRNAs, 356 miRNAs, and 755 proteins in the longissimus dorsi tissues from Sichuan white (SCWrabs) and Germany great line ZIKA rabbits (ZIKArabs). Next, we identified 267 circRNAs, 3 miRNAs, and 29 proteins differentially expressed in the muscle tissues of SCWrabs and ZIKArabs. Interaction network analysis revealed some key regulation relationships between noncoding RNAs and proteins that might be associated with the muscle fiber type and meat quality of rabbit. Further, miRNA isoforms and gene variants identified in SCWrabs and ZIKArabs revealed some pathways and biological processes related to the muscle development. This is the first study of noncoding RNA and protein profiles for the two rabbit breeds. It provides a valuable resource for future studies in rabbits and will improve our understanding of the molecular regulation mechanisms in the muscle development of livestock. More importantly, the output of our study will benefit the researchers and producers in the rabbit breeding program.

## 1 Introduction

As a functional food, rabbit meat can provide properties and be remarkably nutritive (Dalle Zotte and Szendro, 2011). The rabbit industry for meat production is developing rapidly (van Harten and Cardoso, 2010; Paci et al., 2013) and has reached 200 million tons of meat every year, because rabbit meat has characteristics of rich protein, low cholesterol, and low fat. Thus, improving the yield and quality of rabbit meat is important for the breeding program. Sichuan white rabbits (SCWrabs) are a unique Chinese domestic breed which has excellent characteristics, including strong adaptability, fecundity, and resistance to disease, easy to feed, and tender meat (Li S. et al., 2018). ZIKA rabbits (ZIKArabs) are a rabbit breed developed in Germany as a high yielding hybrid for the meat industry and can attend a weight of 3.2 kg in 84 days (Kuang et al., 2014). With our observation, SCWrabs and ZIKArabs have significant differences in the growth rate, adult weight, and meat quality.

Muscle fiber type is important for the muscle growth and meat production of rabbits and the muscle fiber property can be affected by the growth rate (Ruusunen and Puolanne, 2004). It has been reported that many factors, such as age, gender, genetic heritage, muscle function, and production system, can affect the muscle fiber type (Korfage et al., 2009; Park et al., 2009), which can be classified by the isoforms of myosin proteins that are well known for their roles in muscle contraction and intracellular motility (McKoy et al., 1998; Sweeney and Hammers, 2018). In addition, some studies have been demonstrated to identify genes related to the muscle fiber type and meat quality in livestock animals. For example, the skeletal muscle transcriptome study in cattle showed the importance of energy and protein metabolism in determining meat quality after the aging process (Kee et al., 2008); development of longissimus muscle related genes were reported to be different in two different cattle breeds, such as FSTL1, IGFBP5, and FABP5 (Lehnert et al., 2007); MSTN was reported as a regulator of the skeletal muscle development in terms of the lean mass, fat mass, and glucose metabolism (Guo et al., 2009). In rabbit, some genes like FATSO (Xing et al., 2013), MYF5 (Wang et al., 2017a), POU1F1 (Wang et al., 2015), CAST (Wang et al., 2016), and MYPN (Wang et al., 2017b) have been found to be associated with the rabbit meat quality; and Germany great line of ZIKA rabbits (ZIKArabs) were found to be subjected to less growth inhibition from MSTN at slaughter age, which occurred most possibly in skeletal muscles (Kuang et al., 2014). SCWrabs have slower growth rate, lower adult weight, and tender meat, compared to the imported ZIKArabs. However, our knowledge about the molecular basis of muscle fiber type and meat quality of rabbits is still poor, especially for SCWrabs.

Circular RNA (circRNA) and microRNA (miRNA) are two types of regulatory noncoding RNAs. CircRNAs are generated by splicing events occurring during the maturation of the corresponding pre-mRNAs and can be characterized by a closed ring structure without 3 and 5′ ends (Li X. et al., 2018). They have been hypothesized with multiple functions, including regulating the transcription and splicing of their parental genes, acting as miRNA sponges, regulating protein functions through the direct interaction circRNA/protein, and being translated into proteins with a cap-independent mechanism (Greco et al., 2018). It has been shown that circRNAs are abundant and dynamically expressed during muscle development (Greco et al., 2018). In rabbit, a study has identified 9418 circRNAs and seven of them were related to the atherosclerosis (Zhang et al., 2018). miRNAs are small (∼22 nt) noncoding RNAs that can degrade or repress the expression of their target genes (John et al., 2004). Some studies have been demonstrated to identify rabbit miRNAs (Holcomb et al., 2015), however, the rabbit miRNA sequences recorded in miRBase, a database of miRNAs, are much less than other model species like mouse and chicken. More importantly, very little is known about circRNAs and miRNAs in the longissimus dorsi of rabbits.

Transcriptome sequencing has been widely used to study the expression profiles of protein coding and noncoding genes in the muscle development. Some long noncoding RNAs, another type of noncoding RNA, were identified to be differentially expressed in SCWrabs and ZIKArabs and might play important roles in skeletal muscle development (Kuang et al., 2018). Noce and colleagues analyzed the gene expression profile of the ovine skeletal muscle and characterized the genetic variation of transcripts expressed in five Spanish meat sheep breeds (Noce et al., 2018). Sun compared the longissimus muscle transcriptomes of Qianhua Mutton Merino and Small Tail Han sheep (Sun et al., 2016). While iTRAQ (isobaric Tags for Relative and Absolute Quantitation) is a powerful and popular proteomics technology used for protein identification and quantification due to its sensitivity, accuracy, and high throughput (Yang et al., 2016). It has been used to study the protein profiles and functions in multiple tissues from rabbits (Qiu et al., 2011; Zhou et al., 2013; Liu et al., 2015). In the present study, we aimed to use transcriptome sequencing and iTRAQ proteomics to study the difference of circRNAs, miRNAs, and proteins in the longissimus muscle tissues from SCWrabs and ZIKArabs. The output of this study is a valuable resource for future studies and will benefit researchers and producers of rabbits in the breeding program.

## 2 Materials and Methods

### 2.1 Animals

The experiments of this study were conducted based on the guidelines established by the National Institute of Health. The protocols and proposals of this study were approved by The Medical Ethics Committee on Animal Research of the Sichuan Academy of Animal Science. ZIKArabs and SCWrabs were maintained in the experimental area of Sichuan Academy of Animal Science, Sichuan, China. Three male ZIKArabs and three male SCWrabs from the same litter were selected for this project. After they were maintained under the same experimental conditions for 6 months (both reached individual and sexual maturity), the longissimus dorsi tissues were collected and stored in liquid nitrogen for RNA extraction.

### 2.2 Total RNA Extraction

The longissimus dorsi tissue (1 g) of each animal was homogenized in liquid nitrogen, and 10 mg of each homogenized sample was used for total RNA extraction using TRIzol reagent, as described (Chen et al., 2016). In brief, the tissue sample was mixed with 1 ml TRIzol reagent, homogenized using a power homogenizer, and centrifuged (12,000 ×g) for 10 min at 4°C. Then, the fatty layer was removed, and the supernatant was transferred into a new tube. After 0.2 ml chloroform (0.2 ml) was added to the tube and an incubation under room temperature for 3 min, the tube was centrifuged (12,000 × g) for 15 min at 4°C and the aqueous phase was transferred to another RNA tube. For RNA precipitation, we added RNase-free glycogen (10 μg) and 100% isopropanol (0.5 ml) to the tube, and incubated the sample at room temperature for 10 min. After a centrifugation (12,000 ×g, 10 min, 4°C), the RNA pellet was washed using 75% ethanol (1 ml) and air-dried. Then, it was suspended in RNase-free water and water-bathed at 60°C for 10 min. The quality and quantity of the total RNA were determined using the Agilent 2100 Bioanalyzer.

### 2.3 Strand Transcriptome Sequencing With rRNA Removal and Small RNA Sequencing

Total RNA (8 μg) of each sample was processed to remove the ribosomal RNA using the Epicentre Ribo-zero™ rRNA Removal Kit (Epicentre, USA). Then, the rRNA-depleted RNA sample was used for the library construction using the NEBNext^®^ Ultra™ Directional RNA Library Prep Kit for Illumina (NEB, USA), as described (Zhang et al., 2018). Small RNA sequencing libraries of the two rabbit breeds were constructed using the Illumina TruSeq Small RNA Sample Preparation Kit v2, as described (Chen et al., 2019b). Both stranded transcriptome sequencing and small RNA sequencing were performed on the HiSeq 2500 platform in NovoGene (Beijing, China).

### 2.4 Bioinformatics Analysis of circRNAs and miRNAs

We initially cleaned the raw data of transcriptome sequencing and small RNA sequencing using SOAPnuke (Chen et al., 2018). Then, we used “find_circ” to predict the potential circRNAs in each sample according to the protocol and filtered the circRNAs less than 5 reads in at least two replicates (Memczak et al., 2013). To obtain the gene expression profiles of muscle tissues of SCWrabs and ZIKArabs, the clean reads were aligned to the rabbit genome (oryCun2) using HISAT2 and the gene expression was profiled using StringTie, according to the protocols (Pertea et al., 2016). The raw read counts aligned to all genes were measured using htseq-count, as described (Chen et al., 2019a). For miRNA identification, we first aligned the clean reads to the rabbit genome (oryCun2) using SOAP2 software (Li et al., 2009) and predicted the rabbit miRNAs using mireap (https://github.com/liqb/mireap) using the genome mapping results. Next, we aligned the predicted mature miRNA sequences to the miRBase (v22) to identify miRNAs homolog to other species. Both known and novel miRNA sequences were used as the reference for miRNA screen in each sample. miRNAs identified in at least two replicates were kept for downstream analyses. edgeR (Robinson et al., 2010) was used to identify differentially expressed circRNAs, genes, and miRNAs in the muscle tissues of SCWrabs and ZIKArabs with the following cut-offs: log2 fold change (Log2FC) > 1 or < −1, *p*-value < 0.05 and FDR < 0.05.

### 2.5 Protein Preparation and iTRAQ Analysis

Proteins of each sample were prepared from 1 mg muscle tissue, and these were used for the iTRAQ proteomics analysis. The procedures of this experiment were conducted the same as a previous study (Yang et al., 2016). Samples were labelled with the iTRAQ tags as follows: SCWrabs (113, 114, 115) and ZIKArabs (1s16, 117, 118). After the data acquisition was performed on a Q EXACTIVE (Thermo Fisher Scientific, CA, United States) coupled online to the HPLC, we performed the database search and protein quantification using Proteome Discoverer (Thermo Fisher Scientific, CA, United States). For differential protein expression, we first calculated the average abundance of a protein in each group and calculated the ratio using the average protein abundance. Then, t-test was used to calculate the *p*-value. Differentially expressed proteins in the two rabbit groups should satisfy ratio > 1.2 and *p*-value < 0.05.

### 2.6 miRNA, circRNA, mRNA, and Protein Interaction Network

The interactions of miRNA-mRNA and miRNA-circRNA in this study were predicted using the miranda (v3.3a) (John et al., 2004). Then, we predicted the enriched motifs in the mature miRNA sequences using the DREME software (Bailey, 2011) and searched the motifs against the ATtRACT (a database of RNA binding proteins and associated motifs) (Giudice et al., 2016). For circRNAs, we identified their RNA binding proteins (RBPs) using the human data in starBaseV3 (Li et al., 2014). After the miRNA-mRNA, miRNA-circRNA, and circRNA_RBPs were prepared, we visualized their interaction in Cytoscape (v3.7.1).

### 2.7 Mutation Analysis

The mutations in the muscle tissues of SCWrabs and ZIKArabs were characterized using the Strelka pipeline (v2.9.10) (Kim et al., 2018). Then, we removed the variants that passed the filter of Strelka. Next, variants identified in all three replicates were annotated using the ANNOVAR pipeline (Wang et al., 2010) with the Ensembl gene annotation.

## 3 Results

### 3.1 CircRNAs in the Rabbit Muscle

**FIGURE 1:**
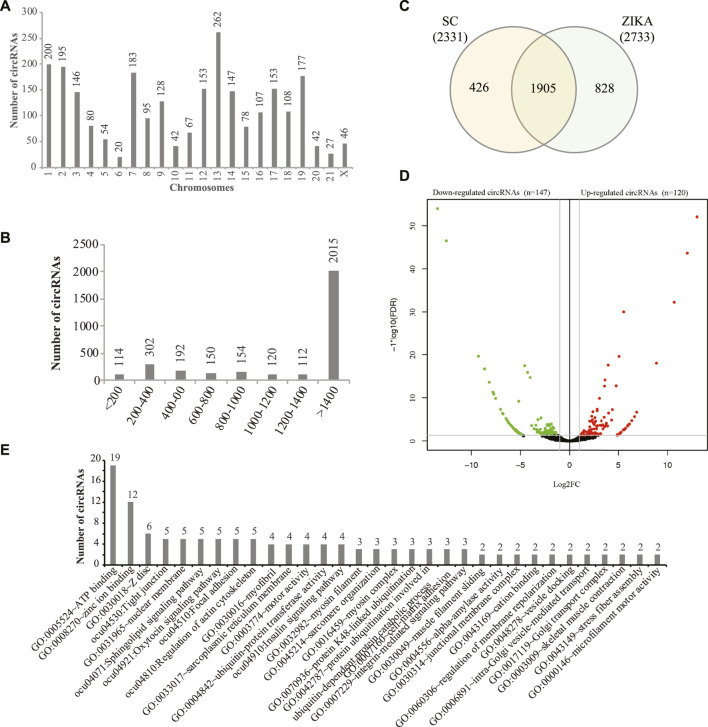
circRNAs identified in the muscle tissue of SCWrabs and ZIKArabs. **(A)** Distribution of circRNAs identified in the rabbit chromosomes. **(B)** Length distribution of circRNAs identified in this study. **(C)** Venn diagram of circRNAs identified in the muscle tissues of SCWrabs and ZIKArabs. **(D)** Volcano plot showing differentially expressed circRNAs in the muscle tissues of SCWrabs and ZIKArabs. **(E)** Functional annotation of the host genes for differentially expressed circRNAs.

Stranded transcriptome sequencing produced 1095.66 million reads (182.61 million reads on average) for all the samples and we obtained 167.37 ∼ 194.56 million reads (12.55 ∼ 14.59 G clean bases) for each sample after data cleaning. Then, the clean data was aligned to the rabbit genome (oryCun2) and it showed that 89.14 ∼ 91.36% of the clean reads mapped. Next, we systematically identified 26,529 circRNAs with 1 or more reads supported in the muscle tissues of SCWrabs and ZIKArabs. After lowly expressed circRNAs (>5 read count in at least two replicates) were filtered, we obtained 3159 circRNAs, of which 2331 and 2733 distributed in SCWrabs and ZIKArabs, respectively (Supplementary Table S1). We showed the numbers of circRNAs identified in all chromosomes of the rabbit genome (Figure 1A) and found that chromosomes chr13, chr1, and chr2 were the top three which can produce circRNAs. Notably, we found that 382 (12.09%), 411 (13.01%), and 854 (27.03%) circRNAs were derived from the intergenic, exon, and intron regions, respectively, and that 1512 (47.86%) circRNAs were formed by both exon and intron parts. Figure 1B showed that 64.95% of the rabbit circRNAs were longer than 1400 bp. Then, we found that 1905 circRNAs were expressed in the muscle tissues of both rabbit breeds and that 426 and 828 circRNAs were specifically expressed in SCWrabs and ZIKArabs, respectively (Figure 1C). Further, we found 31 highly expressed (TPM > 1000) circRNAs in the muscle tissues of two rabbit breeds and that 26 were shared. Using edgeR we identified 120 up-regulated and 147 down-regulated circRNAs in the muscle tissues of SCWrabs compared to ZIKArabs (Figure 1D; Supplementary Table S2). Notably, myosin heavy chain genes are a class of host genes that can derive dysregulated circRNAs, such as MYH1, MYH7, and MYH13, and up-regulated circRNAs in SCWrabs were derived from PI4KA and NEB genes. Functional analysis (Figure 1E) of the circRNA host genes showed that many of them were related to the muscle development related biological processes and pathways, such as “ATP binding,” “Tight junction,” “Regulation of actin cytoskeleton” and “myofibril.”

### 3.2 Gene Expression Profile

**FIGURE 2:**
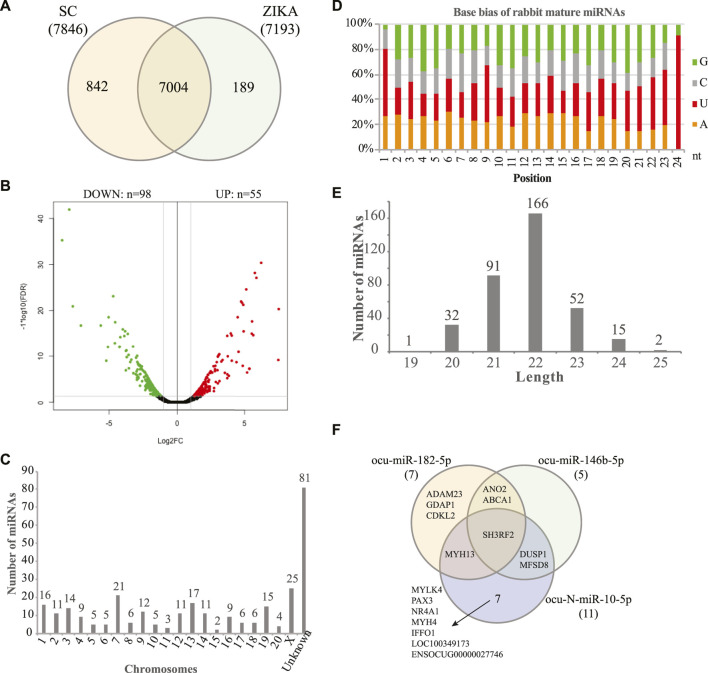
Gene expression profiles and miRNAs identified in the muscle tissues of SCWrabs and ZIKArabs. **(A)** Venn diagram of genes identified in the muscle tissues of SCWrabs and ZIKArabs. **(B)** Volcano plot of differentially expressed genes in the muscle tissues of SCWrabs and ZIKArabs. **(C)** Distribution of miRNAs identified in the chromosomes of rabbit. **(D)** Base bias of the mature miRNAs identified in the muscle tissues of SCWrabs and ZIKArabs. **(E)** Length distribution of miRNAs identified in this study. **(F)** Comparison of target genes for the differentially expressed miRNAs.

Using the sequencing data, we profiled the protein coding gene expression in the muscle tissues of SCWrabs and ZIKArabs. Initially, we identified 7846 and 7193 genes in SC and ZIKA rabbit muscle tissues, respectively. SCWrabs and ZIKArabs shared 7004 genes while 842 and 189 genes were only detected in SCWrabs and ZIKArabs, respectively (Figure 2A). Compared to ZIKArabs edgeR identified 153 differentially expressed protein coding genes (DEGs) in the muscle tissues of SCWrabs (Figure 2B; Supplementary Table S3), including 55 up-regulated and 98 down-regulated genes. It is notable that 4 myosin genes (e.g., MYH4, MYH13, MYLK4, MYL6B) were up-regulated while 9 heat shock protein and 14 histone genes were down-regulated between SCWrabs and ZIKArabs (Table 1). Functional analysis showed 9, 3, and 3 DEGs involved in “negative regulation of cell proliferation,” “negative regulation of growth,” and “fat cell differentiation,” respectively.

**TABLE 1:** Significant gene groups differentially expressed in the muscle tissues of SCWrabs and ZIKArabs.

GeneID	Log2FC	*p*-value	FDR	Regulation	Gene Name	Description
ENSOCUG00000005076	1.48	1.07E-04	4.93E-03	UP	MYLK4	Myosin light chain kinase family member 4
ENSOCUG00000017368	1.29	7.30E-04	2.29E-02	UP	MYL6B	Myosin light chain 6B
ENSOCUG00000010207	1.59	3.38E-05	1.88E-03	UP	MYH13	Myosin heavy chain 13
ENSOCUG00000029652	2.13	4.62E-08	5.15E-06	UP	MYH4	Myosin heavy chain 4
ENSOCUG00000023743	−2.94	2.85E-13	8.37E-11	DOWN		Heat shock 70 kda protein 1B
ENSOCUG00000025112	−3.24	1.94E-15	7.53E-13	DOWN		Heat shock 70 kda protein 1B
ENSOCUG00000001280	−1.82	2.32E-06	1.76E-04	DOWN	HSP90AA1	Heat shock protein 90 alpha family class A member 1
ENSOCUG00000015533	−1.25	1.01E-03	3.00E-02	DOWN	HSPA8	Heat shock protein family A (Hsp70) member 8
ENSOCUG00000004833	−1.98	3.45E-07	3.23E-05	DOWN	DNAJA1	DNAJ heat shock protein family (Hsp40) member A1
ENSOCUG00000014773	−2.06	1.19E-07	1.24E-05	DOWN	HSPH1	Heat shock protein family H (Hsp110) member 1
ENSOCUG00000012690	−1.68	1.26E-05	7.93E-04	DOWN		Heat shock protein family B (small) member 1
ENSOCUG00000027940	−1.91	1.01E-06	8.53E-05	DOWN	HSPA4L	Heat shock protein family A (Hsp70) member 4 like
ENSOCUG00000021817	−4.70	4.68E-27	8.05E-24	DOWN		Heat shock 70 kda protein 1B-like
ENSOCUG00000014459	−1.45	2.72E-04	1.06E-02	DOWN		Histone H3.1
ENSOCUG00000024086	−2.21	3.07E-07	2.90E-05	DOWN		Histone H2A type 1
ENSOCUG00000023129	−1.21	1.79E-03	4.67E-02	DOWN		Histone H2A type 1-E
ENSOCUG00000026810	−2.03	2.45E-05	1.41E-03	DOWN		Histone H2B type 1
ENSOCUG00000027315	−1.54	1.09E-04	5.00E-03	DOWN		Histone H3
ENSOCUG00000027038	−2.70	5.66E-09	7.58E-07	DOWN		Histone H2A type 1
ENSOCUG00000025069	−1.70	2.01E-05	1.19E-03	DOWN		Histone H2A type 1-E
ENSOCUG00000026226	−1.42	9.86E-04	2.94E-02	DOWN		Histone H2A type 1-E
ENSOCUG00000026771	−1.48	3.24E-04	1.21E-02	DOWN	HIST1H2AM	Histone H2A type 1
ENSOCUG00000025038	−1.96	3.53E-05	1.94E-03	DOWN		Histone H3.1
ENSOCUG00000014458	−2.01	1.29E-06	1.06E-04	DOWN		Histone H2A type 1-E
ENSOCUG00000025297	−5.09	2.55E-15	9.32E-13	DOWN		Histone H2A type 1
ENSOCUG00000027227	−1.46	2.73E-04	1.06E-02	DOWN	HIST1H2BB	Histone H2B type 1-B
ENSOCUG00000021035	−2.85	3.03E-11	6.41E-09	DOWN		Histone H2B type 1

### 3.3 miRNA Expression Profiles of Rabbit Muscle Tissues

Using small RNA sequencing, we identified 291 (185 known and 106 novel) miRNA precursor sequences in all the chromosomes of rabbit genome and these miRNA precursors can produce 356 mature miRNAs (Figure 2C; Supplementary Table S3). Among them, 68 were found with both mature and passenger miRNAs (Supplementary Table S3). Bias analysis of nucleotide base showed that the first base preference of rabbit miRNAs was uracil (Figure 2D), and the rabbit miRNAs were enriched in 22 nt in length (Figure 2E). As expected, no specific miRNAs were found in SCWrabs or ZIKArabs. The top 10 highly expressed miRNAs remained the same in the muscle tissues of SCWrabs and ZIKArabs, including ocu-miR-1-3p, ocu-miR-206-3p, ocu-miR-133a-3p, ocu-miR-26b-5p, ocu-miR-101-3p, ocu-miR-378-3p, ocu-miR-27-3p, ocu-let-7d-5p, ocu-let-7i-5p, and ocu-miR-378d-5p. We next identified 3 miRNAs down-regulated in muscle tissues of SCWrabs compared to ZIKArabs (Table 2). Target prediction identified 2124, 2068, and 4694 candidate genes for ocu-miR-182-5p, ocu-miR-146b, and ocu-N-miR-10-5p, respectively. We next analyzed the gene regulation of their targets using the RNA-seq data. It was revealed that 7, 5, and 11 up-regulated genes might be regulated by ocu-miR-182-5p, ocu-miR-146b, and ocu-N-miR-10-5p, respectively (Table 2). It is interesting that some target genes were shared by these three miRNAs (Figure 2F). For example, SH3RF2 was regulated by all three miRNAs; ANO2 and ABCA1 were targeted by ocu-miR-182-5p and ocu-miR-146b-5p; DUSP1 and MFSD8 were shared by ocu-miR-146b-5p and ocu-N-miR-10-5p; and MYH13 was the target gene of ocu-miR-182-5p and ocu-N-miR-10-5p. The regulation relationship between these three miRNAs and their target genes requires further experiments to be explored.

**TABLE 2:** Differentially expressed miRNAs in the muscle tissues of SCWrabs and ZIKArabs.

miRNA	edgeR values	Target number	Gene	Log2FC	FDR	Gene_symbol	Description
ocu-miR-182-5p	Log2FC = -1.40, *p* = 0.0004	2124	ENSOCUG00000010207	1.59	1.88E-03	MYH13	Myosin heavy chain 13
			ENSOCUG00000006775	2.28	1.12E-06	ANO2	Anoctamin 2
			ENSOCUG00000010659	1.51	5.00E-03	ADAM23	ADAM metallopeptidase domain 23
			ENSOCUG00000007700	1.76	5.48E-04	GDAP1	Ganglioside induced differentiation associated protein 1
			ENSOCUG00000003254	1.33	1.74E-02	CDKL2	Cyclin dependent kinase like 2
			ENSOCUG00000009279	1.29	2.15E-02	ABCA1	ATP binding cassette subfamily A member 1
			ENSOCUG00000015667	1.98	4.83E-05	SH3RF2	SH3 domain containing ring finger 2
ocu-miR-146b-5p	Log2FC = -1.23, *p* = 0.0016	2068	ENSOCUG00000006775	2.28	1.12E-06	ANO2	Anoctamin 2
			ENSOCUG00000007999	1.65	1.03E-03	DUSP1	Dual specificity phosphatase 1
			ENSOCUG00000009279	1.29	2.15E-02	ABCA1	ATP binding cassette subfamily A member 1
			ENSOCUG00000015003	1.22	4.34E-02	MFSD8	Major facilitator superfamily domain containing 8
			ENSOCUG00000015667	1.98	4.83E-05	SH3RF2	SH3 domain containing ring finger 2
ocu-N-miR-10-5p	Log2FC = -1.01, *p* = 0.0096	4694	ENSOCUG00000005076	1.48	4.93E-03	MYLK4	Myosin light chain kinase family member 4
			ENSOCUG00000017507	1.49	5.69E-03	PAX3	Paired box 3
			ENSOCUG00000027746	3.68	3.34E-15		
			ENSOCUG00000011746	1.43	7.59E-03	NR4A1	Nuclear receptor subfamily 4 group A member 1
			ENSOCUG00000007999	1.65	1.03E-03	DUSP1	Dual specificity phosphatase 1
			ENSOCUG00000015003	1.22	4.34E-02	MFSD8	Major facilitator superfamily domain containing 8
			ENSOCUG00000015667	1.98	4.83E-05	SH3RF2	SH3 domain containing ring finger 2
			ENSOCUG00000023102	2.17	3.36E-06	LOC100349173	Uncharacterized protein c15orf52 homolog
			ENSOCUG00000010207	1.59	1.88E-03	MYH13	Myosin heavy chain 13
			ENSOCUG00000029652	2.13	5.15E-06	MYH4	Myosin heavy chain 4
			ENSOCUG00000008198	1.29	2.21E-02	IFFO1	Intermediate filament family orphan 1

### 3.4 Protein Expression Profile and Differential Expression

**FIGURE 3:**
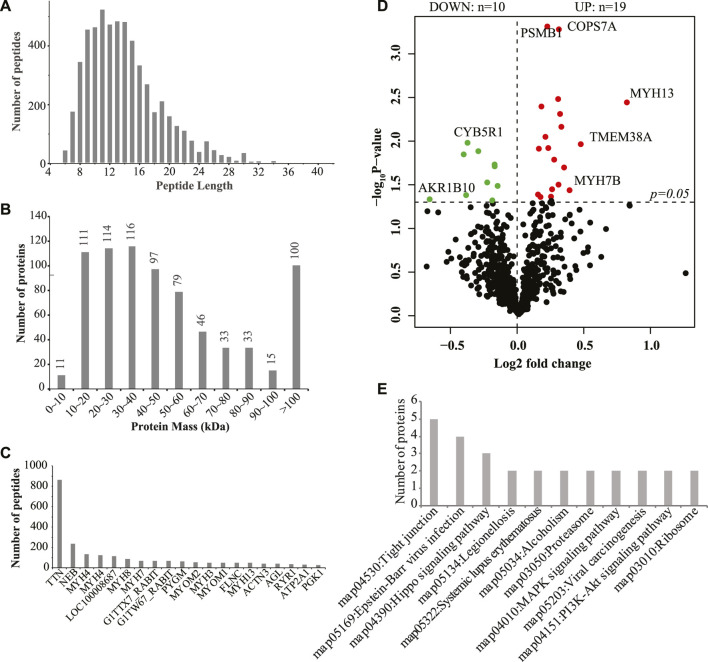
iTRAQ proteomics identified differentially expressed proteins in the longissimus muscle tissues of SCWrabs and ZIKArabs. **(A)** Length distribution of peptides detected in the longissimus muscle tissues by iTRAQ. **(B)** Distribution of protein mass. **(C)** Top highly expressed proteins in the longissimus muscle tissue of rabbits. **(D)** Volcano plot of the differentially expressed proteins in the muscle tissues of SCWrabs and ZIKArabs. **(E)** KEGG pathway enrichment analysis for the differentially expressed proteins.

Then, we used iTRAQ to study the protein profiles of the muscle tissues of SCWrabs and ZIKArabs. Initially, we obtained 269,250 spectra from the liquid chromatography coupled to mass spectrometry (LC-MS/MS) analysis, of which 13,232 (5221 unique peptides) were aligned to 755 rabbit proteins. Length distribution of peptides showed that they were enriched between 9 and 14 amino acids (Figure 3A). The analysis of protein molecular weight (Figure 3B) revealed that 116, 114, 111, and 100 proteins were in 30 ∼ 40 kDa, 20 ∼ 30 kDa, 10 ∼ 20 kDa, and > 100 kDa, respectively. Like miRNAs, no specific proteins were found in the muscle tissues of SCWrabs and ZIKArabs, probably because many rabbit proteins are unknown currently. Next, we showed the top 20 proteins with the most unique peptides identified in the muscle tissues of SCWrabs and ZIKArabs (Figure 3C). TTN was identified with the most unique peptides (865 peptides), followed by NEB (237 peptides) and MYH4 (135 peptides). Differential expression analysis identified 19 up-regulated and 10 down-regulated proteins in the muscle tissues of SCWrabs compared to ZIKArabs (Figure 3D; Table 3). Among them, 3 myosin proteins (MYH7B, MYH8, and MYH13) and 2 proteasome proteins (PSMB1 and PSMC5) were up-regulated in SCWrabs compared to ZIKArabs. Then, we performed KEGG pathway enrichment analysis and found that 5, 3, 2, and 2 proteins were involved by the pathways of “tight junction,” “hippo signaling pathway,” “proteasome,” and “ribosome,” respectively (Figure 3E).

**TABLE 3:** Differentially expressed proteins in the longissimus muscle tissues of SCWrabs and ZIKArabs.

ProteinID	Log2FC	*p*-value	Regulation	Gene name	Description
ENSOCUP00000021805.1	−0.393	0.043	down	AAMDC	Adipogenesis associated Mth938 domain containing
ENSOCUP00000020925.2	−0.667	0.048	down	AKR1B10	Aldo-keto reductase family 1 member B10
ENSOCUP00000006694.2	−0.197	0.050	down	CTNNA1	Catenin alpha 1
ENSOCUP00000014440.2	−0.383	0.011	down	CYB5R1	NADH-cytochrome b5 reductase 1
ENSOCUP00000010958.3	−0.412	0.015	down	HSPD1	Heat shock protein family D (Hsp60) member 1
ENSOCUP00000012421.1	−0.180	0.019	down	LOC100348835	Histone H1.4
ENSOCUP00000007190.2	−0.302	0.014	down	LOC100354435	Heat shock 70 kDa protein 1B
ENSOCUP00000001099.3	−0.157	0.034	down	NNT	Nicotinamide nucleotide transhydrogenase
ENSOCUP00000005290.3	−0.235	0.031	down	USP36	Ubiquitin specific peptidase 36
ENSOCUP00000011737.2	−0.179	0.020	down		
ENSOCUP00000002957.2	0.146	0.043	Up	COPS4	COP9 signalosome subunit 4
ENSOCUP00000009625.2	0.302	0.001	Up	COPS7A	COP9 signalosome subunit 7A
ENSOCUP00000021470.1	0.251	0.037	Up	DHRS7B	Dehydrogenase/reductase 7B
ENSOCUP00000005141.2	0.223	0.012	Up	GPX1	Glutathione peroxidase 1
ENSOCUP00000007030.2	0.243	0.045	Up	H2AFV	H2A histone family, member V
ENSOCUP00000010917.2	0.311	0.005	Up	HSPB1	Heat shock protein family B (small) member 1
ENSOCUP00000021175.1	0.382	0.038	Up	LOC108175352	60S ribosomal protein L17
ENSOCUP00000013763.2	0.201	0.009	Up	LRRC20	Leucine rich repeat containing 20
ENSOCUP00000023874.2	0.812	0.004	Up	MYH13	Myosin heavy chain 13
ENSOCUP00000001968.2	0.341	0.021	Up	MYH7B	Myosin heavy chain 7B
ENSOCUP00000008830.2	0.321	0.007	Up	MYH8	Myosin-8
ENSOCUP00000021074.2	0.171	0.004	Up	PPP2R2A	Protein phosphatase 2 regulatory subunit Balpha
ENSOCUP00000006889.2	0.215	0.001	Up	PSMB1	Proteasome subunit beta 1
ENSOCUP00000003741.2	0.152	0.013	Up	PSMC5	Proteasome 26S subunit, Atpase 5
ENSOCUP00000017814.1	0.165	0.045	Up	SHMT1	Serine hydroxymethyltransferase 1
ENSOCUP00000010672.2	0.466	0.011	Up	TMEM38A	Transmembrane protein 38A
ENSOCUP00000022564.1	0.267	0.017	Up	YWHAG	Tyrosine 3-monooxygenase/tryptophan 5-monooxygenase activation protein gamma
ENSOCUP00000021670.1	0.297	0.003	Up		
ENSOCUP00000025416.1	0.299	0.033	Up		

### 3.5 Noncoding RNA-Protein Interaction Network

To build the interaction network of rabbit noncoding RNAs, genes, and proteins, we first predicted the miRNA targets in rabbit circRNAs and genes. It showed that 3152 circRNAs and 20262 genes can be regulated by the rabbit mature miRNAs. Among them, 129 differentially expressed circRNAs (64 up-regulated and 65 down-regulated) and 160 genes (57 up-regulated and 103 down-regulated) were the potential targets of differentially expressed miRNAs in the muscle tissues of SCWrabs compared to ZIKArabs. Using the iTRAQ results we found 39 protein coding genes (16 up-regulated and 23 down-regulated) regulated by the differentially expressed miRNAs. Next, using the DREME software we found two motifs—AGGUAG and GGAGUCA in 23 and 19 mature miRNA sequences, respectively. In the ATtRACT database only motif AGGUAG was found to interact with 4 (AGO1, DAZAP1, MSI1 and ZRANB2) RBPs. However, if we modify the motifs to AGGUA and GGAGU, 11 (AGO1, DAZAP1, HNRNPA1, HRB27C, KHSRP, MSI1, PTBP1, RBM5, SFPQ, SRSF1, and ZRANB2) and 5 (FMR1, HNRNPDL, LIN28A, MEX-3, and SRSF2) RBPs could be found for AGGUA and GGAGU, respectively. Unfortunately, none of these RBPs were found in the proteomics data. StarBaseV3 identified 18 RBPs binding with 3079 circRNA host genes by similarity. Among them, 19 circRNAs were differentially expressed between the muscle tissues of SCWrabs and ZIKArasb while 2 RBPs were identified in our dataset.

**FIGURE 4:**
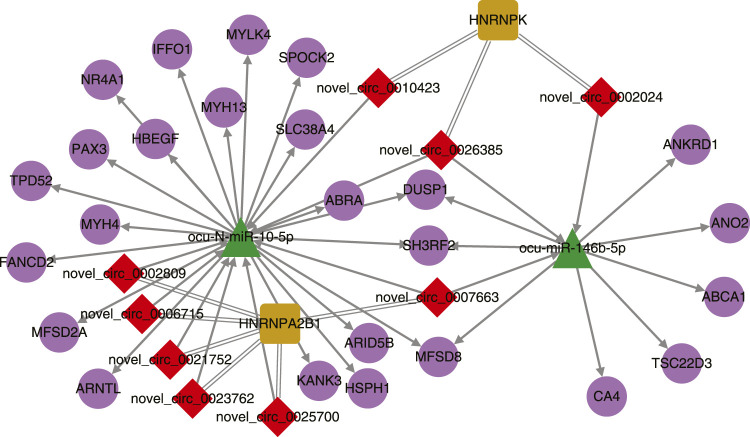
Interaction network of differentially expressed circRNAs, miRNAs, their target genes, and binding proteins.

After simple interactions were filtered, a total of 2 miRNAs, 9 circRNAs, 2 RBPs, and 24 genes were subjected to Cytoscape for visualization (Figure 4). Two miRNAs ocu-N-miR-10-5p and ocu-miR-146b-5p shared 6 targets (3 protein coding genes and 3 circRNAs) while the two RBPs (HNRNPA2B1 and HNRNPK) shared no binding circRNAs. The interactions in noncoding RNAs, protein coding genes, and RBPs require further experiments to be explored in rabbits.

### 3.5 Gene Variants and miRNA Isoforms

Using the small RNA sequencing data, we found that the dominant sequences of 10 miRNAs (6 conserved and 4 novel miRNAs) differed in SCWrabs and ZIKArabs (Table 4). Among them, ocu-N-miR-26-5p was only detected in ZIKA rabbits. Most of these alternations in miRNA sequences could be produced by the cut site preference of DICER enzyme in the cytoplasm because the sequence difference occurred at the end of 3′ arm of miRNA. However, we found a base “A” added to the 5’ arm of ocu-miR-411-5p in SCWrabs, compared to ZIKArabs.

**TABLE 4:** miRNA isoforms identified in the muscle tissues of SCWrabs and ZIKArabs.

miRNA	SCWrabs	Count^a^	ZIKArabs	Count^a^
ocu-miR-29a-3p	CTA​GCA​CCA​TTT​GAA​ATC​AGT​G	96,129,85	CTA​GCA​CCA​TTT​GAA​ATC​AGT	73,85,90
ocu-miR-29d-3p	CTA​GCA​CCA​TTT​GAA​ATC​AGT​G	96,129,85	CTA​GCA​CCA​TTT​GAA​ATC​AGT	73,85,90
ocu-miR-340b-3p	TCC​GTC​TCA​GTT​ACT​TTA​TAG​CC	1,6,9	TCC​GTC​TCA​GTT​ACT​TTA​TAG​C	6,8,5
ocu-miR-411-5p	ATA​GTA​GAC​CGT​ATA​GCG​TAC​G	117,244,252	TAG​TAG​ACC​GTA​TAG​CGT​ACG	119,318,365
ocu-miR-487-3p	AAT​CGT​ACA​GGG​TCA​TCC​ACT​T	13,48,48	AAT​CGT​ACA​GGG​TCA​TCC​ACT	26,35,49
ocu-miR-532-3p	CCT​CCC​ACA​CCC​AAG​GCT​TGC​A	54,81,78	CCT​CCC​ACA​CCC​AAG​GCT​TGC	42,80,98
ocu-N-miR-26-5p	-	0,0,0	TTG​ACC​TAT​GAA​ATG​ACA​GAT​G	6,7,8
ocu-N-miR-48-5p	TTG​CTC​TCT​CTC​TAA​CTC​TGT	41,66,43	TTG​CTC​TCT​CTC​TAA​CTC​TGT​C	32,92,59
ocu-N-miR-79-3p	TCA​GCC​CCT​GGG​GTA​GTT​CTG​A	14,22,30	TCA​GCC​CCT​GGG​GTA​GTT​CTG	17,24,60
ocu-N-miR-80-3p	TCA​GCC​CCT​GGG​GTA​GTT​CTG​A	14,22,30	TCA​GCC​CCT​GGG​GTA​GTT​CTG	17,24,60

a Raw read counts of the miRNA sequence in the three biological replicates.

**FIGURE 5:**
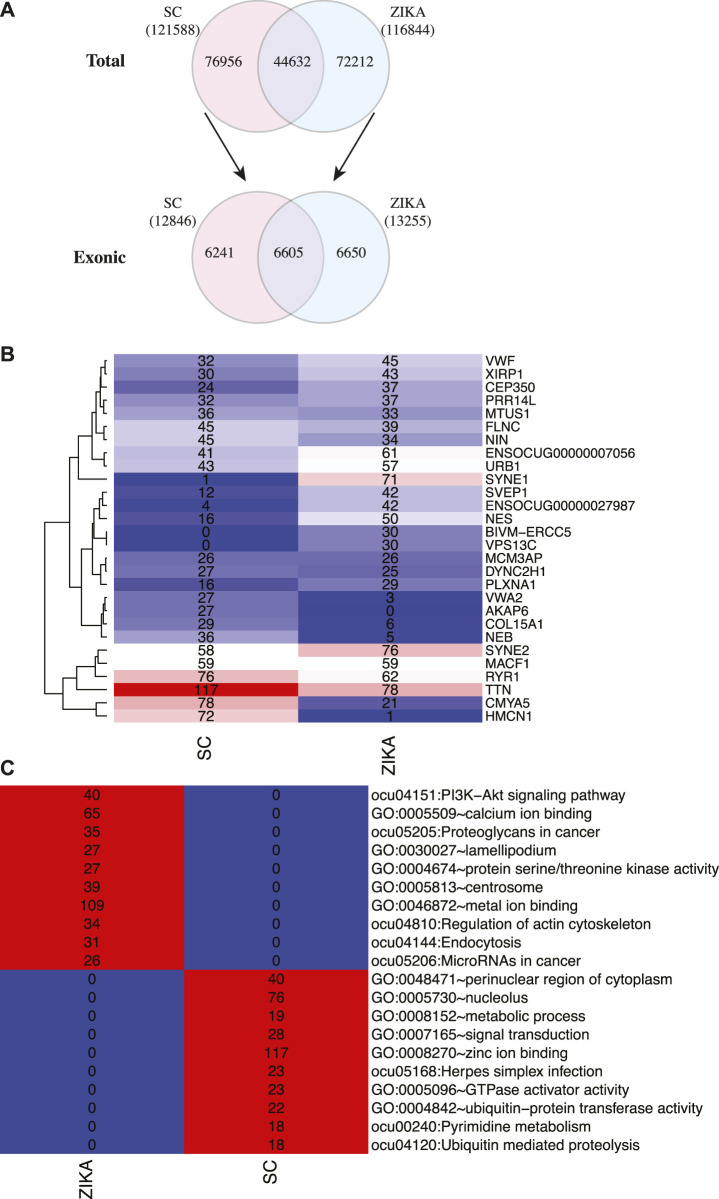
Gene variants identified in the muscle tissues of SCWrabs and ZIKArabs. **(A)** Number of total variants and exonic variants identified in the muscle tissues of SCWrabs and ZIKArabs. **(B)** Number of mutation events in the top 20 genes. **(C)** Functional annotation for the mutated genes specifically identified in the muscle tissues of SCWrabs and ZIKArabs. Values in the blocks represent the numbers of genes involved in the pathways and processes.

Next, we analyzed the variants in protein coding genes using the transcriptome sequencing data. Initially, 121,588 and 116,844 variants were identified in SCWrabs and ZIKA, respectively. After these variants were annotated by ANNOVAR, we found that 6605 exonic variants were shared while 6241 (from 4375 genes) and 6650 (from 4672 genes) exonic variants were specific to SCWrabs and ZIKArabs, respectively (Figure 5A; Supplementary Table 5). It was shown that the top 20 genes with most variants in SCWrabs and ZIKArabs were different (Figure 5B). Interestingly, HMCN1, COL15A1, NEB, VWA2, and AKAP6 were more frequently mutated in SCWrabs, while ENSOCUG00000027987, SYNE1, VPS13C, and BIVM-ERCC5 were more frequently mutated in ZIKArabs. Then, we annotated the specifically mutated genes for SCWrabs and ZIKArabs using DAVID Bioinformatics Resource. After shared terms were filtered, specific pathways and gene ontology terms were shown in Figure 5C. It is notable that muscle development related pathways and biological processes were involved by these specific genes, such as “zinc ion binding,” “regulation of actin cytoskeleton,” “metal ion binding,” “protein serine/threonine kinase activity,” and “calcium ion binding.”

## 4 Discussion

In this study, we identified 3159 circRNAs, 356 miRNAs, and 755 proteins in the longissimus dorsi tissues of two rabbit breeds (SCWrabs and ZIKArabs). They will provide a valuable resource for future research in rabbits. The circRNAs were mainly derived from chromosomes chr13, chr1, and chr2, which agreed with a previous study (Zhang et al., 2018). The features of miRNA sequences identified in this study, such as the first nucleotide preference and length distribution, also agreed with the miRNA database miRBase (Grey et al., 2005). This evidence first supported that the noncoding RNAs identified in this study might be credible. Compared to SCWrabs we identified 267 circRNAs (120 up-regulated and 147 down-regulated), 3 miRNAs (down-regulated), and 29 proteins (19 up-regulated and 10 down-regulated) differentially expressed in the muscle tissues of ZIKArabs and they will be further discussed.

Myosin proteins are motor proteins that convert chemical energy in the form of ATP to mechanical energy, thus generating force and movement (Cooper, 2000). In our study, the transcriptome sequencing and iTRAQ proteomics identified 4 myosin genes (e.g., MYH4, MYH13, MYLK4, MYL6B) (Table 1) and 3 myosin proteins (MYH7B, MYH8, and MYH13) (Table 3), respectively, up-regulated in the muscle tissues of SCWrabs compared to ZIKArabs. In addition, myosin genes (MYH1, MYH7, and MYH13) derived circRNAs were dysregulated in the muscle tissues of SCWrabs compared to ZIKArabs. Also, we found that differentially expressed miRNAs ocu-miR-182-5p and ocu-N-miR-10-5p share the target gene MYH13 and that MYH4 is another target gene of ocu-N-miR-10-5p (Figure 2F). These indicate that myosin and myosin related noncoding RNAs might be one of the reasons for the differences in muscle fiber types and meat qualities of SCWrabs and ZIKArabs. Further, MYH13 is found to be preferentially expressed in most orbital and global fibers in the central innervation zone of rabbit extraocular muscles (Briggs and Schachat, 2002) and it has been proved to colocalize with the MYH4 in global fibers of rabbits (Briggs and Schachat, 2002). In synergy with MYH4 and an increased level of sarcoplasmic reticulum, the localized expression of MYH13 can result in a differentially responsive and very fast contracting region of extraocular muscle (Briggs and Schachat, 2002). The presence of MYH13 and MYH4 in the longissimus dorsi might be related to the good meat quality of SCWrabs. Myostatin (MSTN), also known as growth differentiation factor 8 (GDF-8), is a candidate gene related to the muscle growth. It is a secreted growth factor predominately expressed in skeletal muscle that negatively regulates skeletal muscle mass via the inhibition of myogenesis: muscle cell growth and differentiation (Lee, 2004). It has been shown that the inhibition of MSTN signaling in skeletal muscle can increase the lean mass, reduce the fat mass, and improve the glucose metabolism (Guo et al., 2009). Compared to ZIKArabs, we found a significant upregulation of the MSTN gene in the longissimus dorsi of SCWrabs (Supplementary Table S3). This might explain that SCwrabs have less weight, height, fat mass, and better taste than ZIKArabs.

circRNAs and miRNAs are regulatory RNAs related to the expression of multiple target genes and proteins (Qu et al., 2015; Chen et al., 2019b). They have been shown as key regulators in the muscle development and fiber types. For example, in sheep skeletal muscle 886 circRNAs were identified and many of them (e.g., circ776) interacted with muscle-specific miRNAs which were involved in growth and development of muscle (Cao et al., 2018). In Guizhou miniature pig (*Sus scrofa*), 149 circRNAs were identified to be associated with the muscle growth and the circRNA host genes were found to regulate the muscle development, contraction, chromatin modification, cation homeostasis, and ATP hydrolysis-coupled proton transport (Liang et al., 2017). Further, some circRNA host-genes were found to be related to the myogenesis in the longissimus dorsi of Landrace and Lantang pigs (Sun et al., 2017). In our study, we identified 267 differentially expressed circRNAs in the longissimus dorsi of SCWrabs and ZIKArabs. They might be responsible of the muscle growth and meat quality. In addition, the host genes of some circRNAs were predicted to be involved in the muscle development and fiber type (Figure 1E). However, more experiments are required to validate the association between circRNAs and muscle growth/fiber types in rabbit.

Some miRNAs have been shown to be highly enriched in cardiac and/or skeletal muscle, such as miR-1, miR-133a, miR-133b, miR-206, miR-208, miR-208b, miR-486, and miR-499 (Guller and Russell, 2010). These miRNAs were identified in the current study except miR-486 (Supplementary Table S4). miR-1, miR-133a, and miR-206 were the top three highly expressed miRNAs while miR-208 was lowly expressed (average TPM < 2) in the longissimus dorsi of both SCWrabs and ZIKArabs (Supplementary Table S4). miR-1 can stimulate the myoblast differentiation through the inhibition of HDAC4 (histone deacetylase 4) (Chen et al., 2006); miR-133a increases the myoblast proliferation via the repression of SRF (serum response factor) (Chen et al., 2006); and miR-206 has the influence of the differentiation program via the indirect down-regulation of the helix–loop–helix protein Id, a repressor of MyoD (myogenic differentiation 1) (Kim et al., 2006). We identified three differentially expressed miRNAs in the muscle tissues of SCWrabs compared to ZIKArabs, such as ocu-miR-182-5p, ocu-miR-146b, and ocu-N-miR-10-5p. By targeting NF-B expression, miR-146b promotes myogenic differentiation and modulates multiple gene targets in muscle cells and its homolog miR-146a regulates the maturation and differentiation of vascular smooth muscle cells by targeting NF-κB expression (Dong et al., 2013; Khanna et al., 2014). Interestingly, miR-182 has been studied to inhibit the muscle cell differentiation through the regulation of myogenin (Zhang et al., 2016). It has also been shown to attenuate the atrophy-related gene expression by targeting FoxO3 in skeletal muscle (Hudson et al., 2014). Considering that many miRNAs are unknown for rabbit and the high expression of miRNAs in rabbit longissimus dorsi, it is important to study their associations with the muscle development and fiber type.

In this study, we also identified exonic variants in the muscle tissues of SCWrabs and ZIKArabs (Supplementary Table S5). It is notable that SCWrabs and ZIKArabs had distinct mutation frequency in some genes (Figure 5B). For example, HMCN1, COL15A1, NEB, VWA2, and AKAP6 were more frequently mutated in SCWrabs than ZIKArabs, while ENSOCUG00000027987, SYNE1, VPS13C, and BIVM-ERCC5 were more frequently mutated in ZIKArabs than SCWrabs. Among them, HMCN1 (hemicentin-1), encoding a fibrillar extracellular matrix glycoprotein, is up-regulated in the mice with chronic skeletal muscle fibrosis compared to wild type and up-regulated in the human skeletal muscle in response to oral shilajit supplementation (Das et al., 2016; Chapman et al., 2017), suggesting that HMCN1 might be related to the muscle fibrosis. Also, we found the sequences of mature miRNAs in SCWrabs and ZIKArabs were different (Table 4). Although most of them had the difference at the 3′ arms of miRNA sequences, ocu-miR-411-5p had a base “A” added to the 5’ arm in SCWrabs, compared to ZIKArabs. These miRNA isoforms may result in different target genes in the formation of rabbit longissimus dorsi.

Although this study is the first time to study the profiles of circRNAs, miRNAs, and proteins in the muscle tissues of SCWrabs and ZIKArabs, we have to admit the existence of some limitations and gaps that could be filled by our future research. For example, more samples and timepoints can be applied to compare the difference of muscle development of SCWrabs and ZIKArabs; more data can be generated to support the noncoding RNAs, proteins, and variants that might be related to the muscle development and meat quality of rabbit; and more stringent statistical cut-offs can be used to narrow down the gene candidates.

## 5 Conclusion

In summary, we identified 3159 circRNAs, 356 miRNAs, and 755 proteins in the muscle tissues of two rabbit breeds (SCWrabs and ZIKArabs), of which 267 circRNAs (120 up-regulated and 147 down-regulated), 3 miRNAs (down-regulated), and 29 proteins (19 up-regulated and 10 down-regulated) were differentially expressed. We next performed the interaction network analysis to identify the regulation networks between differentially expressed noncoding RNAs, mRNAs, and proteins in the rabbit muscle tissues. Interestingly, we found 10 miRNAs which had different dominant sequences in the muscle tissues of SCWrabs and ZIKArabs. Further, we analyzed the gene variants which might help explain the genetic background of the two rabbit lines. This is the first time to study noncoding RNA and protein profiles in the muscle tissues of SCWrabs and ZIKArabs. The noncoding RNAs identified in this work can be a valuable resource for future rabbit studies. More importantly, our findings will provide the basis of understanding the molecular mechanisms in the muscle development and differentiation and will benefit the rabbit breeding program.

## Data Availability

The datasets presented in this study can be found in online repositories. The names of the repository/repositories and accession number(s) can be found in the article/Supplementary Material.
